# A computational study of diffusion in a glass-forming metallic liquid

**DOI:** 10.1038/srep10956

**Published:** 2015-06-09

**Authors:** T. Wang, F. Zhang, L. Yang, X. W. Fang, S. H. Zhou, M. J. Kramer, C. Z. Wang, K. M. Ho, R. E. Napolitano

**Affiliations:** 1 Division of Materials Sciences and Engineering, Ames Laboratory, US DOE, Ames, IA 50011, USA; 2 Department of Materials Science and Engineering, Iowa State University, Ames, IA 50011, USA; 3 Department of Physics and Astronomy, Iowa State University, Ames, IA 50011, USA; 4 Kuang-Chi Institute of Advanced Technology, Shenzhen, Guangdong 518000, China

## Abstract

Liquid phase diffusion plays a critical role in phase transformations (e.g. glass transformation and devitrification) observed in marginal glass forming systems such as Al-Sm. Controlling transformation pathways in such cases requires a comprehensive description of diffusivity, including the associated composition and temperature dependencies. In the computational study reported here, we examine atomic diffusion in Al-Sm liquids using ab initio molecular dynamics (AIMD) and determine the diffusivities of Al and Sm for selected alloy compositions. Non-Arrhenius diffusion behavior is observed in the undercooled liquids with an enhanced local structural ordering. Through assessment of our AIMD result, we construct a general formulation for Al-Sm liquid, involving a diffusion mobility database that includes composition and temperature dependence. A Volmer-Fulcher-Tammann (VFT) equation is adopted for describing the non-Arrhenius behavior observed in the undercooled liquid. The composition dependence of diffusivity is found quite strong, even for the Al-rich region contrary to the sole previous report on this binary system. The model is used in combination with the available thermodynamic database to predict specific diffusivities and compares well with reported experimental data for 0.6 at.% and 5.6 at.% Sm in Al-Sm alloys.

The glass forming aluminum-rare-earth (Al-RE) alloys, usually containing more than 85 at.% Al, have attracted considerable interest because of the wide variety of accessible microstructures and the associated range of mechanical properties. For example, Al-RE nanocrystalline composite materials, characterized by fine crystalline phases dispersed in an amorphous or glassy matrix have been shown to exhibit high tensile strength and large strength-to-weight ratio^1^,^2^,^3^,^4^,^5^. These and other novel structures comprised of various stable and metastable phases can be realized through controlled composition and carefully designed rapid solidification and devitrification processes. The Al-Sm binary alloy is of particular interest because of it offers a wide glass forming composition range in the Al-RE series and an array of stable and metastable crystalline phases that are attainable from the liquid or the glass, including fcc, Al_5_Sm (P6/mmm), Al_4_Sm (I4/mmm and Imma) and Al_11_Sm_3_ (Immm), which have all been observed experimentally^1^,^6^,^7^. Navigation of this complex landscape to realize specific phases, structures, and behaviors requires detailed models for the thermodynamic and kinetic properties that govern the material response. The diffusivity of the alloy liquid, particularly in the highly undercooled state, is one such property that is fundamental to understanding phase competition, the glass transition, and microstructural response to various processing conditions^8^ and vitrification^9^.

Experimental values of diffusivity for liquid aluminum and its alloys are scarce^10^. Most direct measurements reported for liquid alloys have used the long-capillary technique and its variations. In this method, convective flow can have a substantial influence on the diffusion profile, and it has been shown that these measurements can overestimate the diffusivity by a factor of 2^11^. A recently developed quasi-elastic neutron scattering (QNS) method^12^ can detect the microscopic dynamics at the atomic length scale and with picosecond resolution. At this time scale, convective flow can be neglected, and accurate self-diffusivity can be deduced^11^,^13^. This method was employed by Demmel *et al*.^14^ and by Kargl *et al*.^15^ to measure the temperature-dependent self-diffusivity of liquid Al, and the two set of data are in good agreement. Specifically, an activation energy of *Q* = 27.0 ± 6.8 kJ/mole was determined from the experimental data^15^, associated with the Arrhenius behavior,


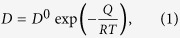


where *R* is the gas constant, *T* the absolute temperature, and *D*^0^ is a pre-exponential factor.

Self-diffusivity *D* in the liquid may also be determined indirectly from the shear viscosity η with the use of Stokes-Einstein relation,


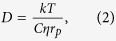


where *k* is the Boltzmann constant, and *r*_*p*_ the radius of a particle moving in the fluid. The constant *C* is determined by the hydrodynamic boundary condition for the fluid on the particle surface, varying from 6π for the sticking boundary condition to 4π for the slipping boundary condition. The available experimental viscosity data of liquid aluminum have been reviewed by Assael *et al*.^16^. Using their recommended values^16^, the QNS results^14^,^15^ of liquid Al can be well-reproduced by Eq. 2 with the slipping boundary condition.

The only experimental investigation of diffusivity in Al-Sm liquids have been reported by Wang and his colleagues^17^, however their original data^18^ remain unpublished. Wang *et al*.^17^ proposed a non-Arrhenius description for diffusion coefficient of Al-Sm liquids: 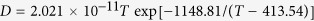, and the composition dependence is assumed negligible. Their results will be discussed in the following section.

A complimentary approach is to determine diffusivities by molecular dynamics (MD) simulations, which is a widely used for investigating the structural and dynamic properties in the liquid state^19^,^20^. With a potential accurately describing the atomic interactions, MD can provide a comprehensive description of the single-atom as well as the collective behavior. The Einstein relation is widely used in analysis of MD results, relating the self-diffusion coefficient to the mean square displacement (MSD) as


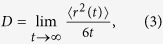


where *r* indicates the atom position, which is a function of observation time *t*. This approach has been applied to Al^10^,^21^, Ni^22^, Al-20 at.% Ni^23^ and Ni-5.4 at.% W^24^ melts using ab initio potentials, and the results are always in good agreement with the experimental data.

The literature results of self-diffusion coefficients in liquid Al are summarized in Fig. 1. Self-diffusion coefficients in liquid Al have been studied extensively by classical MD using the semiempirical embedded-atom method (EAM) formalism, and the results are strongly dependent on the different implementations of the EAM potential^25^. Alfe and Gillan^21^ reported a self-diffusion coefficient in liquid Al of 6.8 × 10^−9^ m^2^/s at 1000 K by using ab initio molecular dynamics (AIMD), which agrees with the experimental data^14^,^15^. Jakse and Pasturel^10^ studied dynamic properties of liquid aluminum using AIMD within the local-density (LDA) and generalized gradient (GGA) approximations, and they found the GGA approximation enhances the icosahedral short range order (ISRO) and then provides slightly higher activation energy and lower diffusivity.

In previous work by several of the current authors^26^, AIMD simulations were performed at 1500, 1300, 1100 and 900 K to investigate the structural evolution in Al_90_Sm_10_ liquid, and diffusivities of Al and Sm were derived from the mean square displacements for the above temperatures. The obtained diffusivity data can be approximated by the Arrhenius relationship within the simulation uncertainties. A more comprehensive AIMD study is performed in this work by including several different alloy compositions with multiple temperatures selecting from both the above-melting and the undercooling regions.

In the present work we seek a comprehensive model to describe the diffusivities in Al-Sm liquids over a range of compositions and temperatures. We start from the general approach suggested by Andersson^27^, building a phenomenological diffusion model on the basis of atomic mobility *M*_*i*_ of diffusional elements *i*:


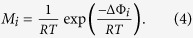


For diffusion in a disordered solution phase, the composition and temperature dependence of the generalized activation energy ΔΦ_*i*_ can be expressed by Redlich-Kister polynomials^28^


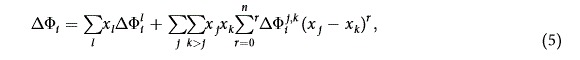


where *x* indicates the mole fraction of elements. 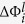 and 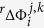 are model parameters. This treatment offers particular utility in enabling the use of kinetic databases for the modeling of diffusion in multicomponent systems, where standard parameterization permits calculation of self/tracer diffusivity, intrinsic chemical diffusivity, and interdiffusion coefficients for alloys. The self/tracer diffusion coefficient *D*_*i*_ obtained from MD simulations can be connected with *M*_*i*_ by


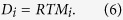


Furthermore, the mobility database can formulated to be used in conjunction with a standard CALPHAD database, offering a comprehensive picture of the thermodynamic and kinetic landscape^29^.

## Results and Discussion

### Self and tracer-diffusivity in liquid Al and Sm

**Figure 1:**
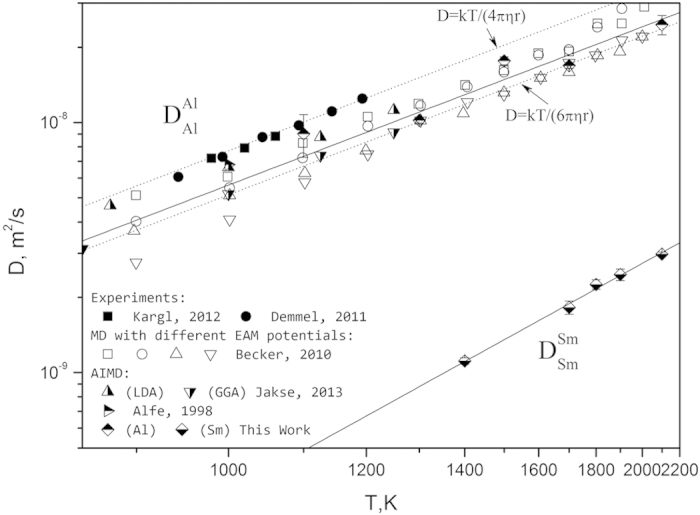
Self-diffusion coefficients in liquid Al and liquid Sm. The solid symbols are experimental data^14^,^15^ for Al and the open symbols are classical MD results for Al with different EAM potentials^25^. The half-solid diamonds and triangles indicate AIMD results from this work and the literature^10^,^21^, respectively.

The self-diffusivity of Al in liquid Al obtained from this work has been presented in Fig. 1, comparing with data from experiments^14^,^15^ and MD simulations^10^,^21^,^25^. As shown in Fig. 1, the results from different sources are in general agreement with each others. The overall temperature dependence from experiments (*Q* = 27.0 ± 6.8 kJ/mole^14^,^15^) has been well-captured by both classical MD (*Q* = 24.9 ~ 29.9 kJ/mole^25^) and AIMD (*Q* = 24.2 kJ/mole, this work), and the absolute values of the diffusion coefficient have been slightly underestimated by MD simulations with a factor of 1.2 ~ 1.7 (1.35 for the present AIMD results), which demonstrates the capability of MD simulations in describing the structural and mass transport properties of liquid Al. The self-diffusion coefficients of Sm in liquid Sm from AIMD are also plotted in Fig. 1, which can be approximated by the Arrhenius equation with an activation energy of 35.0 kJ/mole. We note here that our results for Al and Sm are consistent with the widely observed correlation between melting temperature and activation energy for self-diffusion, as summarized empirically by Iida’s relation^30^:


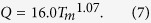


According to this relation, the activation energy for self-diffusion in liquid-Al and liquid-Sm are 24.1 and 35.6 kJ/mole, respectively, which are in good agreement with our values (i.e. 24.2 and 35.0 kJ/mole, respectively).

**Figure 2:**
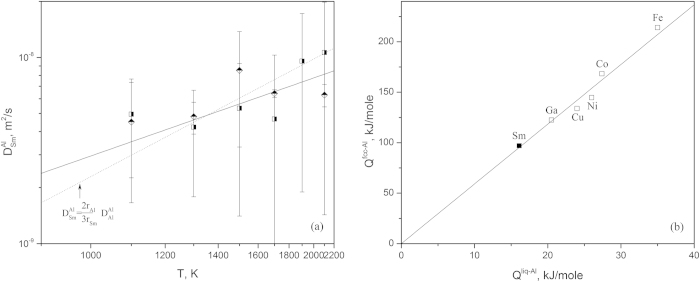
Tracer diffusivity of Sm in liquid Al (a) by considering an empirical correlation between the activation energy for diffusion of impurities in solid and liquid Al (b). The half-solid symbols in (a) indicate AIMD data for the dilute alloys from this work of two specific cases: (i) 1 at% solution (one Sm atom with 99 Al atoms) by square symbols and (ii) the pure Al limit computed by extrapolation by diamond symbols.

The impurity diffusion coefficient of Sm in liquid Al is plotted in Fig. 2a. From a statistical point of view, the impurity diffusion coefficient from AIMD simulations is not accurate and then the associated uncertainty is very large as shown in Fig. 2a, which makes it difficult to derive a reliable mobility description for tracer diffusion from AIMD results unless additional restrictions can be introduced. Gorecki^31^,^32^ connected the activation energy for diffusion in the liquid state with that in the solid state by considering the liquid phase as a strongly defected crystal. He studied the changes of activation energy of impurity diffusion in Ag, Cu^31^ and Fe^32^ when the system passes the melting point, and found that the ratio of activation energy between the solid and liquid states is around 5 (specifically, 5.35 for Ag, 5.12 for Cu and 4.63 for Fe). Here we tested this correlation for Al in Fig. 2b. The activation energy values of diffusion were taken from Du *et al*.^33^, who reviewed the experimental data for diffusion of some solutes in fcc and liquid Al. According to their paper^33^, only the impurity diffusivities of Cu, Ni, Fe, Co and Ga in liquid Al have been subjected to systematic measurements and then been used by the present work. An inspection of those elements confirmed the simple relation suggested by Gorecki^31^,^32^ and the average ratio is 5.92 for these solutes in Al.

Returning now to the issue of Sm diffusion in the Al liquid, we use the correlations discussed above to reexamine the AIMD data for the dilute alloys. Here we use the term “dilute” to indicate two specific cases employed using a 100-atom AIMD simulation scheme: (i) 1 at % solution (one Sm atom with 99 Al atoms), and (ii) the pure Al limit computed by extrapolation. By including this ratio into assessment, the fit for 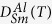 shown by the solid line in Fig. 2a is determined by a least-squares method, and the optimized parameters are listed in Table 1.

As an alternative to the correlation employed in the treatment described above, we offer here another approach that can be achieved by using the Stokes-Einstein relation, which asserts that the ratio of solute diffusivity in the dilute alloy to that of the pure solvent scales with the ratio of their respective atomic radii. If the tracer diffusivity of Sm in the Al liquid follows the Stokes-Einstein relation and the sticking boundary condition is applied due to the large solute atom, we have


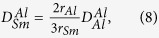


which gives the dotted line in Fig. 2a with a higher activation energy than the prvious approach (24.2 vs. 16.1 kJ/mole). To test this observation, we analyze here the experimental results of U diffusion in the Al liquid due to the similar sizes between Sm and U atoms. Mitamura *et al*.^34^ measured the impurity diffusivity of U in liquid Al from 1073 K to 1283 K and derived an activation energy of 19.7 kJ/mole from their data, and the extrapolation yields a value in agreement with the result from cathodic deposition in liquid metal/electrolyte systems at 973 K^35^. Similar to what we discussed above for Sm, the diffusivity data of U in the Al liquid can also been reasonably reproduced by the Stokes-Einstein relation with the sticking boundary condition, and the activation energy (24.2 kJ/mole) is higher than the one from the numerical fitting (19.7 kJ/mole).

No AIMD simulation has been performed for the impurity diffusion of Al in liquid Sm because the interest of this work is focused on Al-rich part and AIMD is not well suited to provide accurate impurity diffusivity as mentioned above. The tracer diffusivity of Al in the Sm liquid has been estimated from the self-diffusivity in the Sm liquid in this work. Tracer diffusion of solute particles in a simple solvent has been studied by Ould-Kaddour and his coworkers^36^,^37^ with solvent and tracer molecules interacting through Lennard-Jones potentials. The diffusivity ratio between solute and solvent were reported to be dependent on their size ratio and their mass ratio. A microscopic calculation of the tracer diffusion coefficient of a small tagged particle in a dense liquid of much larger particles were performed by Bhattacharyya and Bagchi^38^, and the solute motion was coupled to both the collective density fluctuation and the transverse current mode of the liquid. Their results for a wide range of solute–solvent size ratio are in good agreement with Ould-Kaddour’s work^36^. Based on their results, we assumed 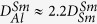 in this work.

### Diffusion modeling for liquid Al-Sm alloys

**Figure 3:**
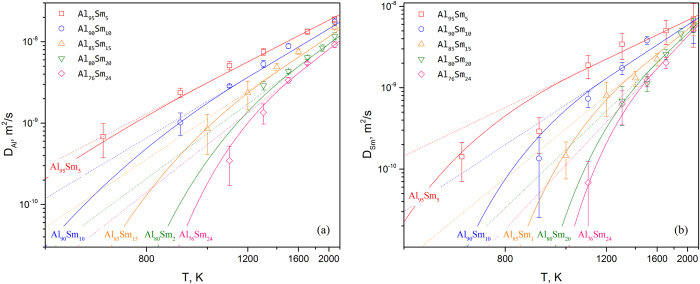
Calculated self-diffusion coefficients of Al (a) and Sm (b) in five liquid Al-Sm alloys. The solid and dotted lines indicated calculations with and without VFT corrections for the undercooling temperature range, respectively. Symbols show AIMD results from the present work.

To derive the associated composition and temperature dependencies, the AIMD data were assessed in the terms of a diffusion model suggested by Andersson^27^. The assessed mobility parameters for liquid Al-Sm alloys are listed in Table 1 and the calculated self/tracer diffusion coefficients of Al and Sm in selected Al-Sm alloys are plotted by dotted lines in Fig. 3a,b, respectively. By comparing with AIMD results in Fig. 3, we found that most high-temperature (*T *> *T*_*L*_, where TL is the liquidus temperature) data can be well reproduced by the calculation and negative deviations from the calculated values are observed for the low-temperature (*T *< *T*_*L*_) region. By inserting Eq. 4 into Eq. 6, Eq. 1 is reproduced for 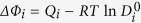. In another word, the diffusion model from Andersson^27^ is based on an Arrhenius behavior. However, as shown in the present work, the diffusion coefficient can be generally approximated by the Arrhenius equation only at high temperatures while off-Arrhenius behavior of diffusivity was observed in the undercooled state, which is also reported in previous MD investigations^39^,^40^,^41^,^42^. In order to understand the off-Arrhenius slowdown in the undercooled liquid, we investigated the local structure development in the liquid and observed a rapid growth of local clusters (short-range ordering) in the undercooled state. Similar observation has been reported^43^ for Al450Sm50 alloy with a large simulation cell of 500 atoms. Since those local clusters are energetic favorable^43^, one can expect atoms in those clusters are less mobile, and then the rapid enhancement of short-range order in the undercooled liquid will slow down the dynamics, making the deviation of diffusivity from the Arrhenius equation.

To extend the mobility database (Table 1) into the low-temperature range, a Volmer-Fulcher-Tammann (VFT) equation^44^,^45^ is adopted in this work:


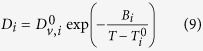


where *D*_*v*,_^0^_*i*_, *B*_*i*_ and *T*_*i*_^0^ are parameters evaluated from the fitting process. Here we developed a connection between the mobility database with the VFT description to reduce the number of fitting parameters in Eq. 9, and then to avoid overfitting caused by the lack of accurate low-temperature data. By assuming the diffusion behavior switching from Arrhenius type to VFT type at *T*_*L*_, due to the continuity of *D* and its first derivative, we get


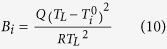


and


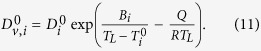


Once 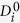 and *Q* are determined from the mobility database, the number of fitting parameters of the VFT equation can be reduced to one by considering Eqs. 10 and 11.

With low-temperature (*T *< *T*_*L*_) region described by VFT formula, the complete description of self/tracer diffusivity in liquid Al-Sm alloys is presented by solid lines in Fig. 3a,b. Almost all AIMD results are reproduced within the simulation uncertainties. The model parameters for VFT description of different Al-Sm alloys are given in Table 2.

### Interdiffusivity in liquid Al-Sm alloys

**Figure 4:**
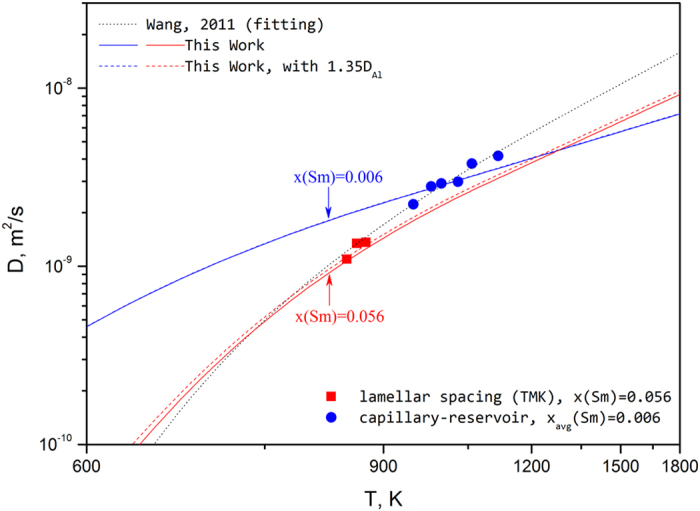
Calculated chemical diffusion coefficients for Al-0.6 at.% Sm and Al-5.6 at.% Sm alloys in comparison with the experimental estimation^17^,^18^.

Using the complete diffusion description developed in this work, the interdiffusion coefficients for liquid Al-Sm alloys of different compositions have been calculated and are plotted by the solid lines in Fig. 4. As shown in Fig. 4, the composition dependence of diffusivity is quite strong, even for the Al-rich region, which is contrary to Wang’s assumption that composition dependence is small^17^. The dotted line represents the fitting of experimental data suggested by Wang *et al*.^17^, which shows an obvious deviation from our calculations. We think the deviation is mainly caused by the composition dependence ignored by Wang’s fitting^17^. To test this hypothesis, we contacted the authors of Ref.^17^ for their original data and experimental details. Wang *et al*.^18^ used a capillary reservoir technique, measuring composition profiles to determine the interdiffusion coefficient for selected temperatures in the range from 948 K to 1116 K, which provides high-temperature data for their fitting. We note here, for clarity, that the measured composition profiles suggest an average composition of 0.6 at.% Sm. They also studied the eutectic spacing variation in Al-5.6 at.% Sm^17^ using laser scanning technique. According to TMK model^46^, for eutectic growth at high velocities, the eutectic spacing variation is strongly influenced by the temperature-dependent diffusion coefficient. Therefore, the diffusion coefficients in undercooled Al-Sm melt were estimated^17^,^18^ from the connection between eutectic spacing and velocity, which gives the low-temperature data for their fitting. In general, as shown in Fig. 4, both set of experimental data can be well reproduced by the present calculation when the corresponding compositions are taken into account. Based on their high-temperature data obtained by the capillary reservoir technique, Wang *et al*.^18^ reported a stronger temperature dependent (*Q* = 32.4 kJ/mole) than the present assessment (*Q* = 17.4 kJ/mole). It should be mentioned here that the long-capillary measurements are easily affected by convective flow^11^,^12^. As Meyer and Kargl indicated in their paper^11^,^47^, the self-diffusivity in liquid Cu from long-capillary measurements^48^ shows a stronger temperature-dependence than the results from QNS^13^. It is also mentioned by Wang *et al*.^18^ that the activation energy derived from their capillary reservoir measurements contains a large deviation, which may not provide a proper description of the temperature dependence of the diffusion coefficient. As shown in Fig. 4, the diffusivity data for undercooled Al-5.6 at.% Sm liquid are slightly higher than the present calculated values. On one hand, those data were estimated from the eutectic spacing variation measurement with many assumptions^46^, and then a large uncertainty is expected. On the other hand, as we mentioned before, the self-diffusion coefficient of Al in liquid Al have been slightly underestimated by AIMD simulations with a factor of 1.35. By correcting the diffusivity of Al with a factor of 1.35, a better reproduction of those experimental data can be achieved as shown by the dashed line in Fig. 4.

## Summary

In this work, we studied diffusion in liquid Al-Sm alloys by combining AIMD simulations and diffusion modeling. First, we computed diffusion coefficients for Al and Sm in liquid Al95Sm5, Al90Sm10, Al85Sm15, Al80Sm20 and Al76Sm24 alloys by means of AIMD simulations from the above-melting temperature range to the undercooling temperature range. Non-Arrhenius behavior of diffusivity was observed for all five alloys as they are cooled below their melting temperatures, which is likely caused by the local structural ordering. By using those diffusivity data, we constructed a diffusion mobility database for liquid Al-Sm alloys that includes composition and temperature dependence. The non-Arrhenius diffusion behavior observed in the undercooled liquid was described by a VFT equation and a connection between the mobility database with the VFT description was made to reduce the number of fitting parameters. Using the model, which is based on AIMD simulation results, we computed diffusivities for selected compositions and compared directly to independent experimental reports. This comparison (for 0.6 at% Sm and 5.6 at% Sm) shows that the model predicts reasonably well the observed behavior.

## Methods

Initially, six model alloys containing 100 atoms (i.e. Al99Sm1, Al95Sm5, Al90Sm10, Al85Sm55, Al80Sm20 and Al76Sm24) were chosen for the AIMD simulations for deriving self/tracer diffusion coefficients for different alloy compositions. The general investigation interest is focused on Al-rich part, where a complex competition between various crystalline phases and the amorphous phase in the quenching process has been suggested by experimental observations^1^,^49^,^50^. The AIMD simulations for this system were performed using the Vienna ab initio simulation package (VASP)^51^. The generalized gradient approximation^51^,^52^ for the exchange-correlation energy was used and the interaction between ions and valence electrons was described by the projected augmented-wave method (PAW)^53^. A cubic unit cell containing 100 atoms was used with periodic boundary conditions. The simulations were performed in the NVT ensemble (constant number of atoms, constant volume and constant temperature) with Nose thermostats. The MD time step was set to 3 fs and the Verl*et al*gorithm was employed to integrate Newton’s equations of motion. Only the Γ-point was used to sample the Brillouin zone. The samples were first prepared at 2100 K to reach thermal equilibrium (well above the liquidus temperature *T*_*L*_,), followed by cooling with a rate of 100 K per 1000 MD steps to 4-5 selected temperatures including both above-*T*_*L*_ temperatures and below-*T*_*L*_ temperatures. The pressure of the system at each temperature was tuned to zero by adjusting the size of the cubic simulation cell. After the system was thermally equilibrated at each temperature, an additional 12000 MD steps (36 ps) are followed to collect the atomic trajectories for the analysis. The mean square displacements as a function of time (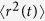) were calculated at different temperatures, and the self/tracer diffusion coefficients were derived using the Einstein relation (Eq. 4). Additional AIMD simulations were performed for liquid Sm to determine the generalized activation energy 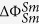.

Secondly, the high-temperature (*T *> *T*_*L*_) diffusivities were collected from AIMD simulations, from which a diffusion mobility database were constructed based on the modeling provided by Andersson and Agren^27^ and Engstrom *et al*.^29^. After that, the low-temperature (*T *< *T*_*L*_) diffusivity data were included to determine the parameters *T*_0*i*_ in the VFT equation. Eqs. 10 and 11 have been considered into the fitting process and *T*_0*i*_ was assumed to be a linear function of composition in the Al-rich region. And then, the diffusion database for liquid Al-Sm has been developed.

At last, the chemical diffusivities were calculated from our diffusion database for selected Al-Sm alloys for a comparison with results from Wang *et al*.^17^,^18^. A recent thermodynamic database for Al-Sm system^54^ was selected to provide thermodynamic quantities, e.g. derivatives of the chemical potential, for this work.

## Additional Information

**How to cite this article**: Wang, T. *et al*. A computational study of diffusion in a glass-forming metallic liquid. *Sci. Rep*. **5**, 10956; doi: 10.1038/srep10956 (2015).

## Figures and Tables

**Table 1:** Assessed mobility parameters for liquid Al-Sm alloys, (J/mole).

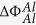	24206 + 133.731 × T
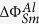	16058 + 147.224 × T
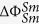	35005 + 146.434 × T
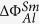	35005 + 139.879 × T
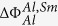	99039 − 14.933 × T
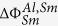	218087 − 93.193 × T

**Table 2:** VFT Model parameters for liquid Al-Sm of different compositions.

x(Sm)	*T*_*L*_, K	*T*_*Al*_^0^, K	*B*_*Al*_	*D*_*v*,_^0^_*Al*_, m^2^/s	*T*_*Sm*_^0^, K	*B*_*Sm*_	*D*_*v*,_^0^_*Sm*_, m^2^/s
0.05	1067	146	2640	6.889 ×10^−8^	384	1346	1.148 ×10^−8^
0.10	1227	260	2554	5.542 ×10^−8^	470	1720	1.377 ×10^−8^
0.15	1443	375	2535	5.059 ×10^−8^	556	2124	1.928 ×10^−8^
0.20	1655	489	2520	4.801 ×10^−8^	641	2471	2.675 ×10^−8^
0.24	1624	580	2228	3.663 ×10^−8^	710	2303	2.284 ×10^−8^
